# A fossil species of the enigmatic early polypod fern genus *Cystodium* (Cystodiaceae) in Cretaceous amber from Myanmar

**DOI:** 10.1038/s41598-017-14985-7

**Published:** 2017-11-03

**Authors:** Ledis Regalado, Alexander R. Schmidt, Marc S. Appelhans, Bork Ilsemann, Harald Schneider, Michael Krings, Jochen Heinrichs

**Affiliations:** 1 Ludwig Maximilian University, Faculty of Biology, Department of Biology and Geobio-Center, Menzinger Straße. 67, 80638 Munich, Germany; 2 Instituto de Ecología y Sistemática, Carretera de Varona 11835 e/ Oriente y Lindero, La Habana 19, CP 11900, Calabazar, Boyeros, La Habana, Cuba; 3 University of Göttingen, Department of Geobiology, Goldschmidtstraße 3, 37077 Göttingen, Germany; 4 University of Göttingen, Albrecht-von-Haller Institute for Plant Sciences, Department of Systematics, Biodiversity and Evolution of Plants, Untere Karspuele 2, 37073 Göttingen, Germany; 5 SNSB-Bayerische Staatssammlung für Paläontologie und Geologie, Richard-Wagner-Straße 10, 80333 München, Germany; 6 Center for Integrative Conservation, Xishuangbanna Tropical Botanical Garden, Menglun, Mengla, 666303 Yunnan, China; 7 Natural History Museum, Department of Life Science, London, SW75BD UK; 8 Ludwig Maximilians University, Department of Earth and Environmental Sciences, Palaeontology and Geobiology, Richard-Wagner-Straße 10, 80333 München, Germany

**Keywords:** Palaeontology, Plant evolution

## Abstract

The monospecific fern genus *Cystodium* (Cystodiaceae; Polypodiales) occurs exclusively in the tropical forests of the Malay Archipelago, the Admiralty Islands, the Louisiade Archipelago, and the Solomon Islands. Divergence time estimates suggest that the genus originated in the Mesozoic; however, fossil evidence to validate this suggestion has been lacking. Amber from Myanmar (Burmese amber) is an important source of new information on the diversity of vascular cryptogams in the Cretaceous. This paper describes the fossil taxon *Cystodium sorbifolioides* nov. sp. based on a fragment of a fertile leaf preserved in Burmese amber that represents the first fossil evidence of the family Cystodiaceae. *Cystodium sorbifolioides* is used to obtain a minimum age estimate for the Cystodiaceae and the closely related, monogeneric Lonchitidaceae and Lindsaeaceae. The fossil strengthens the hypothesis that the forest ecosystems of Malesia and Melanesia represent refugia for many tropical plant lineages that originated in the Cretaceous.

## Introduction

The enigmatic, early polypod fern genus *Cystodium* J. Sm. (Cystodiaceae; Lindsaeineae, Polypodiales) as presently defined contains only one species, *C. sorbifolium* (Sm.) J. Sm., which thrives exclusively in the tropical forests of the Malay Archipelago, the Admiralty Islands, the Louisiade Archipelago, and the Solomon Islands^1,2^. This region is not only home to some of most spectacular and species-rich tropical forest ecosystems^3,4^, but is also known as the habitat of the four extant members of the once widespread and diverse fern family Matoniaceae, which is believed to have had a near-global distribution in the Mesozoic era^5^. However, the Malesian region, as we know it today, has been formed relatively recently, during the Miocene, as a result of the collision of the North Australian Craton with Southeast Asia^6,7^. The ancestors of many of the plants occurring in the Malesian region today are found in Asia or Australia where they evolved in the tropical or boreo-tropical forests of the global greenhouse during the late Paleocene and early Eocene^8,9^. The genus *Cystodium* is arguably one of these Cenozoic relict taxa that originated during the Cretaceous^10–13^. Its present-day geographic distribution is likely the result of a range expansion facilitated by the formation of the Malesian Archipelago during the Miocene.

The systematic position and classification of *Cystodium sorbifolium* has been a matter of debate since the taxon was initially described. Until relatively recently, the taxon was mostly regarded as belonging to the family Dicksoniaceae M. R. Schomb. based on indusium morphology and because older individuals resemble small tree ferns^1,2^. However, these considerations did not take into account several lines of morphological evidence that clearly argued against affinities of *C. sorbifolium* to the Dicksoniaceae, including the grooved upper surface of the petiole, cylindrical sorus receptacles, and the near-vertical position of the sporangium annulus^1,14,15^. Based on these structural features, the genus was excluded from the Dicksoniaceae by Croft^15^ and transferred to a new family, Cystodiaceae J. R. Croft. Moreover, this author^15^ noted similarities of *Cystodium* to the Dennstaedtiaceae s.l. with regard to soral structure. However, this notion was not accepted^2^. Application of DNA sequence variation eventually provided evidence for close affinities of *Cystodium* to basal polypod lineages such as the Lindsaeaceae C. Presl ex M. R. Schomb.^16–18^. The most recent classification of ferns places *Cystodium* in a separate family, Cystodiaceae, nested in the suborder Lindsaeineae together with the families Lindsaeaceae and Lonchitidaceae Doweld^19^.

Divergence time estimates have variously been used to suggest an origin of *Cystodium* in the Early Cretaceous (113.0–153.0 Ma)^12^, Jurassic (167.1 Ma)^11^ or even Triassic (180–220.5 Ma)^13^. However, the Cystodiaceae represent a ghost lineage that lacks a documented fossil record, which could be used to corroborate its presence in the Mesozoic. Over the last years, fossils of fern foliage^20–23^ exquisitely preserved in mid-Cretaceous amber from Myanmar^24–27^ have become increasingly important as a source of new information on Cretaceous fern diversity and have contributed greatly to the resolution of several striking discrepancies between the age estimates obtained by molecular dating and the minimum ages of lineages provided by the fossil record, for instance with regard to the crown group Dennstaedtiaceae Lotsy^21^. Moreover, the recent discovery of a fossil assignable to the Lindsaeaceae in Burmese amber^22^ provided evidence of the presence of early diverging polypod ferns of the suborder Lindsaeineae in the Cretaceous. Although this find is in agreement with divergence time estimates in molecular phylogenetic frameworks^10–13,21^, the fossil has not yet been used to calibrate a dated phylogenetic hypothesis for its putative extant relatives.

In this study, we describe the fossil taxon *Cystodium sorbifolioides* nov. sp. based on a fragment of a fertile leaf preserved in Burmese amber. This discovery is important because it represents the first piece of macrofossil evidence of the existence of the Cystodiaceae in the mid-Cretaceous. Moreover, it suggests the coexistence in the Cretaceous forests of Myanmar of fossil members of the families Cystodiaceae and Lindsaeaceae, both within the suborder Lindsaeineae. The newly described fossil is used to obtain a minimum age estimate for the fern family Cystodiaceae, as well as for the closely related Lonchitidaceae and Lindsaeaceae. This estimate is compared with results obtained from estimates in which either only the unnamed Lindsaeaceae fossil has been used^22^ or both fossils are used together to calibrate a phylogenetic hypothesis for the extant members of the suborder Lindsaeineae and the closely related Saccolomatineae. Finally, the new fossil validates the hypothesis that the forest ecosystems of Malesia and Melanesia represent refugia for many tropical plant lineages that originated in the Cretaceous.

## Results

### Systematic Paleontology

#### Classification

Eukaryota, Viridiplantae, Streptophyta, Embryophyta, Tracheophyta, Moniliformopses, Polypodiidae, Polypodiales, Lindsaeineae, Cystodiaceae, *Cystodium*.

**Figure 1:**
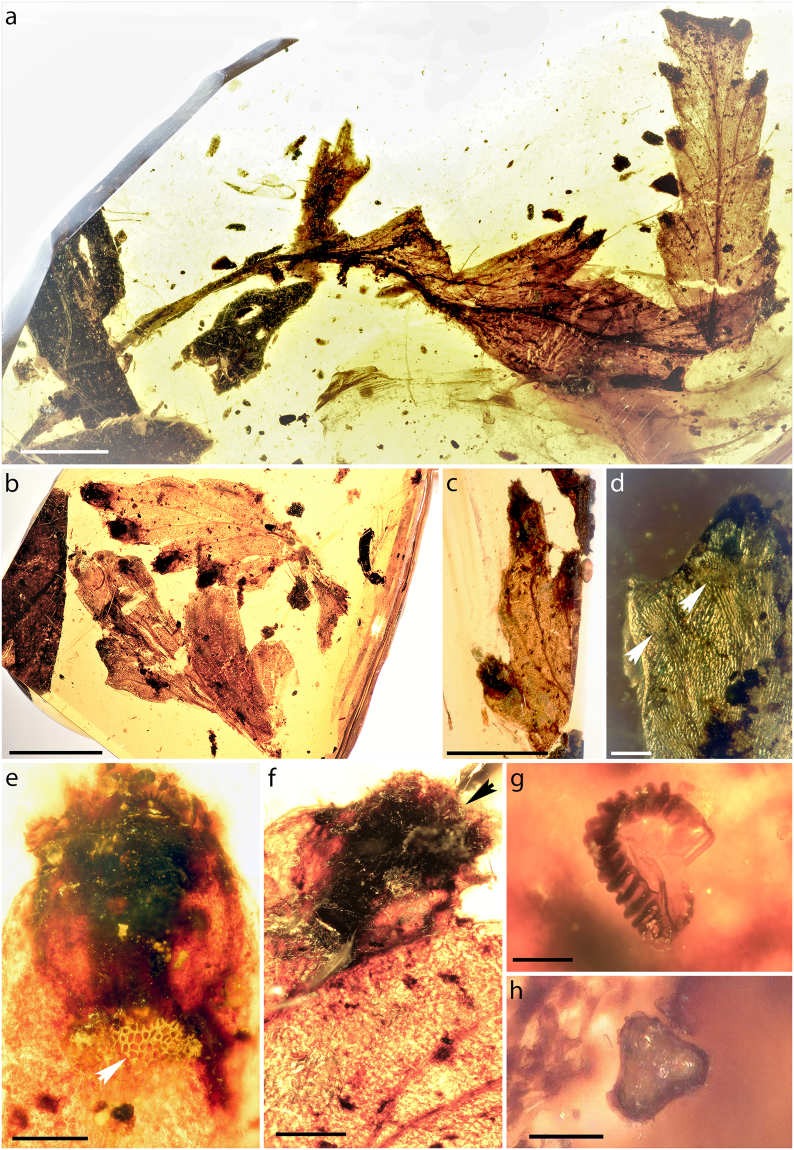
Fossil of *Cystodium sorbifolioides* in Burmese amber (GZG.BST.21964). (**a**) Holotype; large leaf fragment (GZG.BST.21964a). (**b**,**c**) Smaller leaf fragments (GZG.BST.21964b,c). (**d**) Adaxial surface; arrowheads indicate widened tips of veins and polygonal cells in outer indusia (GZG.BST.21964b). (**e**,**f**) Abaxial blade showing marginal sori terminal on veins. White arrowhead indicates polygonal cells in basis of outer indusium (GZG.BST.21964b). (**f**) Marginal sorus. Black arrowhead shows paraphyses (GZG.BST.21964b). (**g**) Empty sporangium with vertical annulus (GZG.BST.21964b). (**h**) Polar view of trilete spore (GZG.BST.21964b). Scale bars: 3 mm (**a**,**b**,**c**), 300 µm (**d**,**e**,**f**), 100 µm (**g**), 50 µm (**h**).

**Figure 2:**
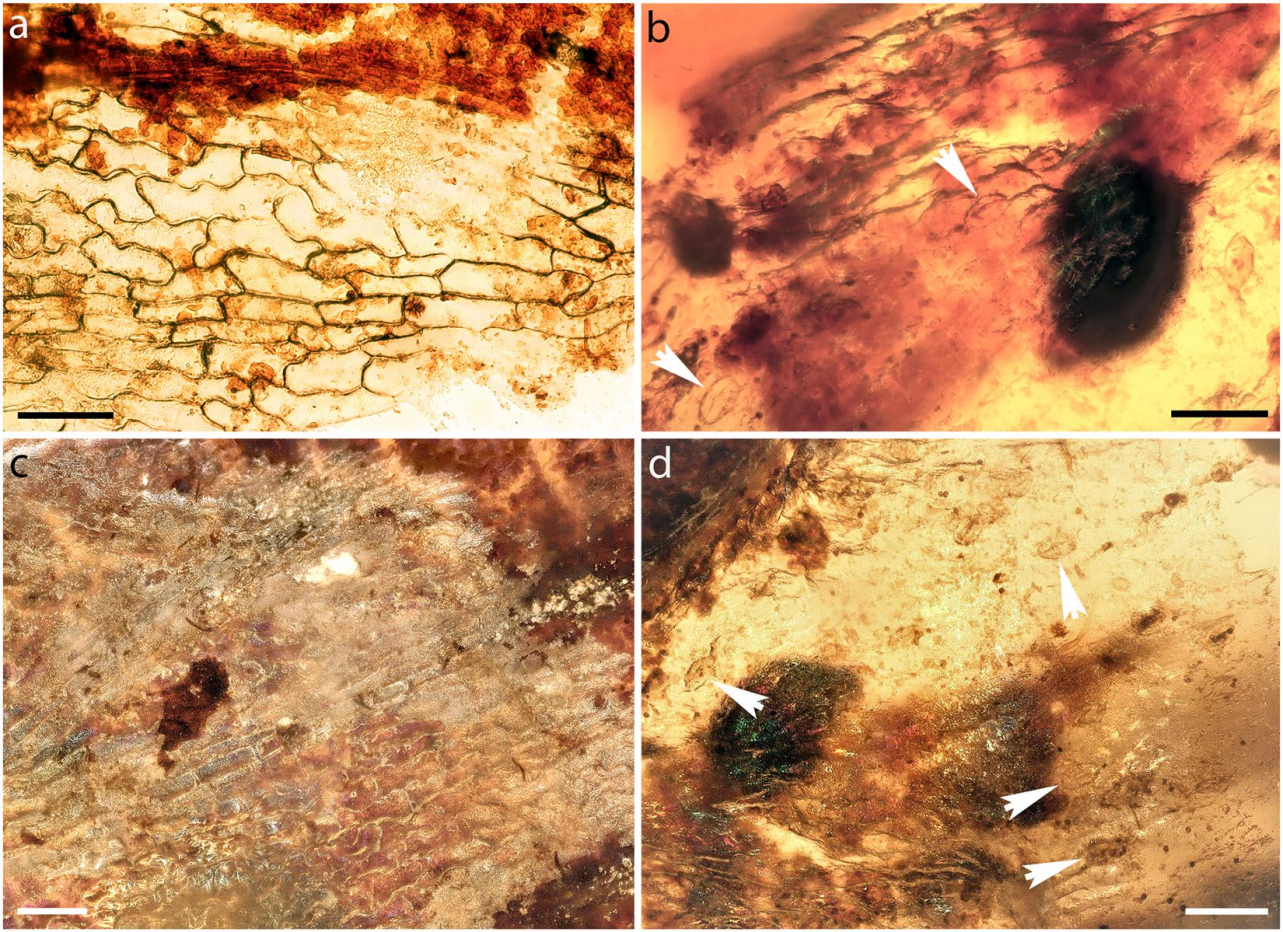
Epidermis of *Cystodium sorbifolioides* in Burmese amber (GZG.BST.21964b). (**a**) Adaxial epidermis showing cells with weakly undulate anticlinal walls. (**b**–**d**) Abaxial epidermis showing epidermal cells with weakly sinuous anticlinal walls. White arrowheads in b and d indicate anomocytic stomata. Scale bars: 100 µm (**a**–**d**).

*Cystodium sorbifolioides* nov. sp. L.Regalado, A.R.Schmidt, H.Schneid., M.Krings et Heinrichs (Fig. 1a–h; Fig. 2a–d).

#### Holotype

Fern inclusion in Burmese amber piece GZG.BST.21964a, Fig. 1a.

#### Type locality

Amber mines near Tanai, Ledo Road, 105 km northwest of Myitkyina, Kachin State, Myanmar (26°20’N, 96°36’E). This site occurs within the Hukawng Basin, which is comprised of folded sedimentary (volcanic) rocks of Cretaceous and Cenozoic age^25^.

#### Type horizon

Burmese amber (Burmite)^24,25^, Earliest Upper Cretaceous, earliest Cenomanian, absolute age 98.79 ± 0.62 million years ago established by U-Pb dating of zircons from the rind of the unprocessed amber^26^.

#### Etymology

The specific epithet refers to the striking morphological resemblance of the fossil to foliage of the extant *Cystodium sorbifolium*.

#### Diagnosis

Pinna axes adaxially grooved. Pinna segments (or pinnules) entire-margined or blunt-dentate; venation free, catadromous, secondary veins 1-forked (rarely 2-forked); pinnae (or pinnules) hypostomatic, glabrous, epidermis of both sides composed of elongate cells with weakly undulate anticlinal walls; stomata anomocytic. Sori apical, marginal, one per segment, terminal on broadening vein tips; outer indusium formed by lamina, slightly curved, inner indusium concave, receptacle not projecting; epidermis of outer indusium composed of polygonal, almost isodiametric cells with thickened anticlinal walls. Sporangia with vertical annulus interrupted by stalk; abundant pluricellular paraphyses with clavate tips occur between sporangia. Spores tetrahedral-globose, trilete, approximately 30–60 μm in size, with non-prominent ornamented perine.

#### Description

Three portions of blades of this fern occur in a single block of Burmese amber (4.2 × 1.9 cm) that has subsequently been cut into three pieces to allow better photographic documentation of the individual inclusions. The blade of this fern was at least pinnate-pinnatifid (probably 2-pinnate). Pinnae (or pinnules) are up to 3.8 cm long and 7 mm wide. Axes are adaxially grooved. Individual pinnule segments are up to 5 mm long and 7 mm wide, with the margins mostly entire or 2–3 blunt-dentate at apex. The venation is free and catadromous, with the secondary veins forking once (rarely twice) in their course to the margin. Pinnae (or pinnules) are hypostomatic. The epidermis of the adaxial and abaxial sides is composed of elongate cells, 80–200 × 15–60 μm in size, that are regularly arranged parallel to the main vein and characterized by weakly undulate anticlinal walls. Pinnae (or pinnules) and axes appear to be glabrous (based on the areas where the epidermis is preserved). Stomata are 40–55 long, 20–30 μm wide, and anomocytic. Sori are apical, marginal, one per segment, and located terminally on the broadening tips of the lateral veins. They are wide tubular or conical in shape and covered by a slightly curved, outer indusium formed by the pinna (or pinnule) lamina, and a second, concave, inner indusium that is attached at least at the base and 0.7–1 × 0.5–0.7 mm in size. The receptacle is up to 530 μm long and does not project. The epidermis of the outer indusium consists of distinctly polygonal, almost isodiametric cells, which are 10–30 × 12–30 μm in size and possess thickened anticlinal walls. Sporangia attain sizes of 180–220 μm, and are characterized by a near-vertical annulus that is interrupted by the sporangium stalk. Abundant pluricellular paraphyses with clavate tips occur interspersed with the sporangia. Spores are tetrahedral-globose, trilete, approximately 30–60 μm in size, and possess an inconspicuously ornamented perine.

### Divergence time estimates

**Figure 3:**
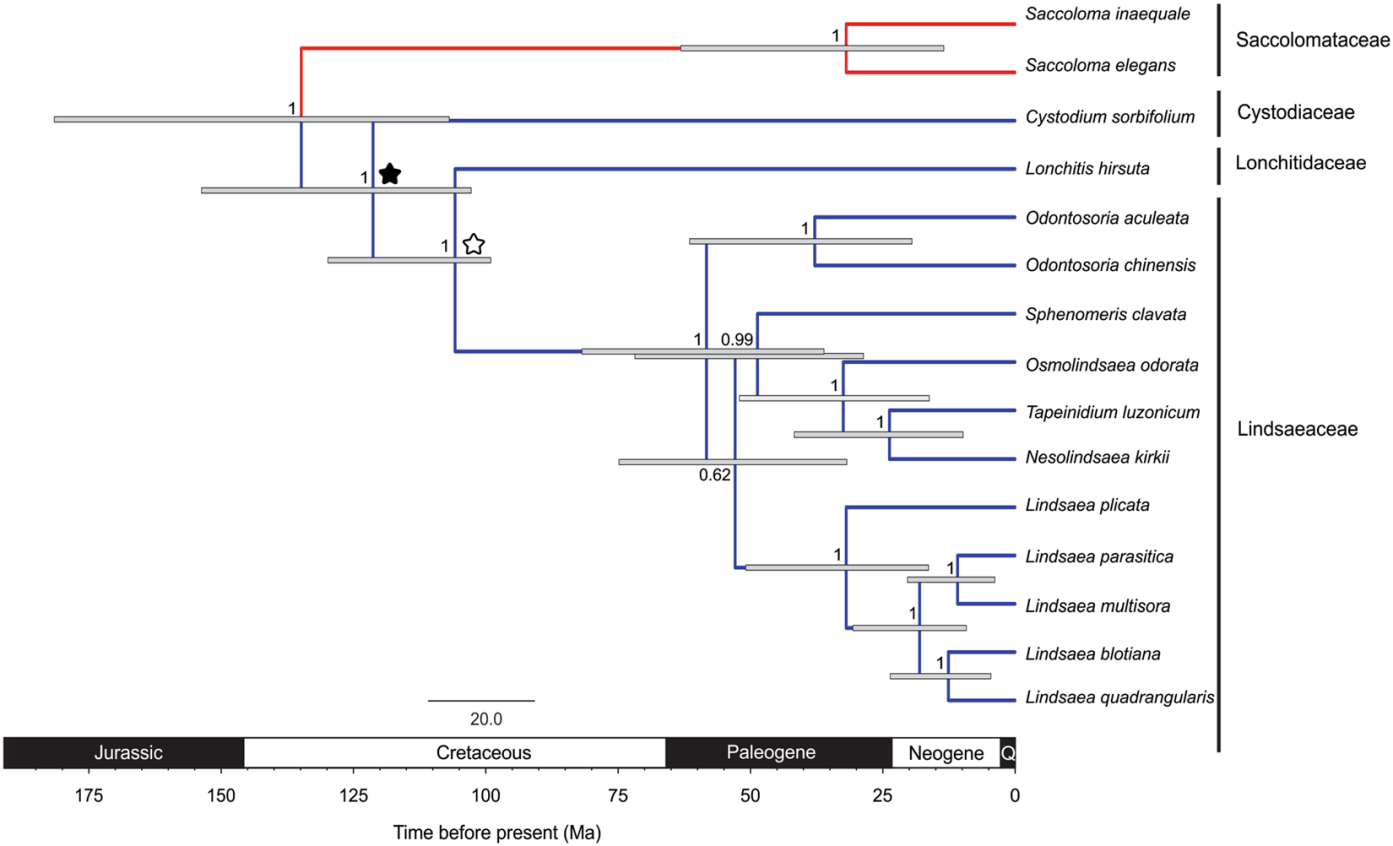
Time-calibrated phylogeny of early polypods in the suborders Saccolomatineae and Lindsaeineae based on five plastid markers. Gray bars indicate 95% highest posterior density for each node and numbers shown above the branches are posterior probabilities. Red clade represents suborder Saccolomatineae and blue clade represents suborder Lindsaeineae. Black asterisk shows calibration point using as minimum bound the age of *C. sorbifolioides*. White asterisk indicates calibration point using as minimum bound the age of unnamed Lindsaeaceae fossil^22^.

The divergence time estimates obtained by applying three different approaches are suggestive of a diversification of the Lindsaeineae crown group some time between the Early Jurassic and Late Cretaceous, and Saccolomatineae between the Late Cretaceous and Paleogene-Miocene (Table 1, Fig. 3). Narrower intervals were acquired when the *Cystodium* fossil described in this study, as well as the unnamed fossils of Lindsaeaceae mentioned in the Introduction section of this paper, were included in the analysis (Fig. 3), while the youngest ages were obtained by including only the new *Cystodium* fossil.

**Table 1:** Comparison of divergence time estimates of the polypod fern suborders Saccolomatineae and Lindsaeineae obtained from the following approaches: (1) assignment of the fossil *Cystodium sorbifolioides* to the split between Cystodiaceae and Lonchitidaceae-Lindsaeaceae, (2) assignment of the unnamed Lindsaeaceae fossil^22^ as minimum age of the split between Lonchitidaceae and Lindsaeaceae and (3) assignment of both *Cystodium sorbifolioides* and Lindsaeaceae fossils.

Family/Suborder		Approach 1	Approach 2	Approach 3	Published estimates^12^	Published estimates^11^	Published estimates^13^
Cystodiaceae		111.2 [98.8–162.0]	131.8 [102.7–197.5]	121.0 [102.5–153.3]	134.0 [113.0–153.0]	167.1	206.4 [180.0–220.5]
Lindsaeaceae	crown	52.5 [30.4–81.8]	63.2 [35.4–98.4]	58.2 [36.0–81.6]	49.0 [40.0–60.0]	47.0 [42.4–62.6]	182.3 [162.9–184.4]
	stem	96.2 [68.8–146.6]	111.4 [98.8–163.6]	105.5 [98.8–129.5]	121.0 [103.0–140.0]	151.0	247.1 [241.0–252.5]
Lonchitidaceae		96.2 [68.8–146.6]	111.4 [98.8–163.6]	105.5 [98.8–129.5]	121.0 [103.0–140.0]	151.0	206.4 [180.0–220.5]
Saccolomataceae	crown	30.4 [11.9–59.8]	35.7 [14.5–73.7]	31.8 [13.4–62.0]	7.3 [5.0–10.0]	—	35.3 [35.2–36.8]
	stem	124.4 [99.3–187.8]	147.5 [107.6–230.8]	134.5 [106.6–181.1]	143.0 [123.0–167.0]	179.9	236.1 [227–239.5]
Saccolomatinae-Lindsaeinae		124.4 [99.3–187.8]	147.5 [107.6–230.8]	134.5 [106.6–181.1]	143.0 [123.0–167.0]	179.9	247.1 [241.0–252.5]

Published estimates^11–13^. In square brackets values of 95% highest posterior density intervals. All values given in mybp. Approach (3) shown in Fig. 3.

## Discussion

### Systematic position

Members in the order Polypodiales, which comprises >80% of extant fern diversity, are characterized by sporangia with a vertical or near-vertical ring of annulus cells^19,28,29^. The occurrence of sporangia with a (near-) vertical annulus in the fossil *Cystodium sorbifolioides* is therefore indicative of systematic affinities of this fern to the Polypodiales. Precisely as in the extant *Cystodium sorbifolium*, however, annulus orientation in the fossil *C. sorbifolioides* does not entirely coincide with that seen in typical representatives of Polypodiales. Tetrahedral trilete spores are common in all the six families of non-eupolypod Polypodiales (i.e. Saccolomataceae Doweld, Cystodiaceae, Lonchitidaceae, Lindsaeaceae, Pteridaceae E. D. M. Kirchn. and Dennstaedtiaceae^28–30^).

Within the Saccolomataceae, only a single genus, *Saccoloma* Kaulf., is currently recognized^19^. This taxon differs from the fossil described in this study in that the blade is anadromously dissected^31^, the acroscopic sides of all divisions are larger than the basiscopic sides, and the ultimate segments are asymmetrical at base^32^. Members of Lindsaeaceae producing trilete spores (*Lindsaea* Dryand. p.p., *Nesolindsaea* Lehtonen & Christenh*., Odontosoria* Fée and *Sphenomeris* Maxon) also are distinct from the fossil in that they possess an anadromously dissected lamina^33^. Moreover, the sori typically occur on the uniting ends of several veins (*Lindsaea* p.p. and *Nesolindsaea*). Although *Odontosoria* and *Sphenomeris* generally share with the fossil the formation of sori on single veins at segment apices (one per segment), they differ from the latter in having strongly anadromous blades with the ultimate divisions usually connected to each other by a narrow wing of tissue^33^. Ferns in the families Lonchitidaceae and Pteridaceae with marginal or intramarginal sori are distinguished from the fossil by having elongate sori that are protected by the reflexed pinnule margin, rather than a true indusium^28,31,34^.

Six genera within the family Dennstaedtiaceae, namely *Dennstaedtia* Bernh.*, Leptolepia* Mett. ex Prantl*, Microlepia* C. Presl*, Monachosorum* Kunze*, Oenotrichia* Copel. and *Pteridium* Gled. ex Scop.^28,30,31,35^, produce tetrahedral trilete spores, precisely as seen in the fossil. However, two of the three Dennstaedtiaceae lineages^21,35^ differ from the fossil with regard to soral morphology. The genus *Monachosorum* is exindusiate^21,36^, while *Pteridium* is indusiate, but characterized by elongate marginal sori covered by a false outer and true, rudimentary inner indusium. Sori that are morphologically similar to those seen in the fossil occur in *Dennstaedtia* and its relatives, *Leptolepia, Microlepia*, and *Oenotrichia*^21,31,35,37^. However, this lineage of the Dennstaedtiaceae possesses marginal or submarginal sori with cup- to pouch-shaped or subreniform indusia lacking paraphyses^31,35,37^, with the sole exception of *Oenotrichia*, which is characterized by submarginal sori with receptacular appendages protected by subreniform indusia^31^.

**Figure 4:**
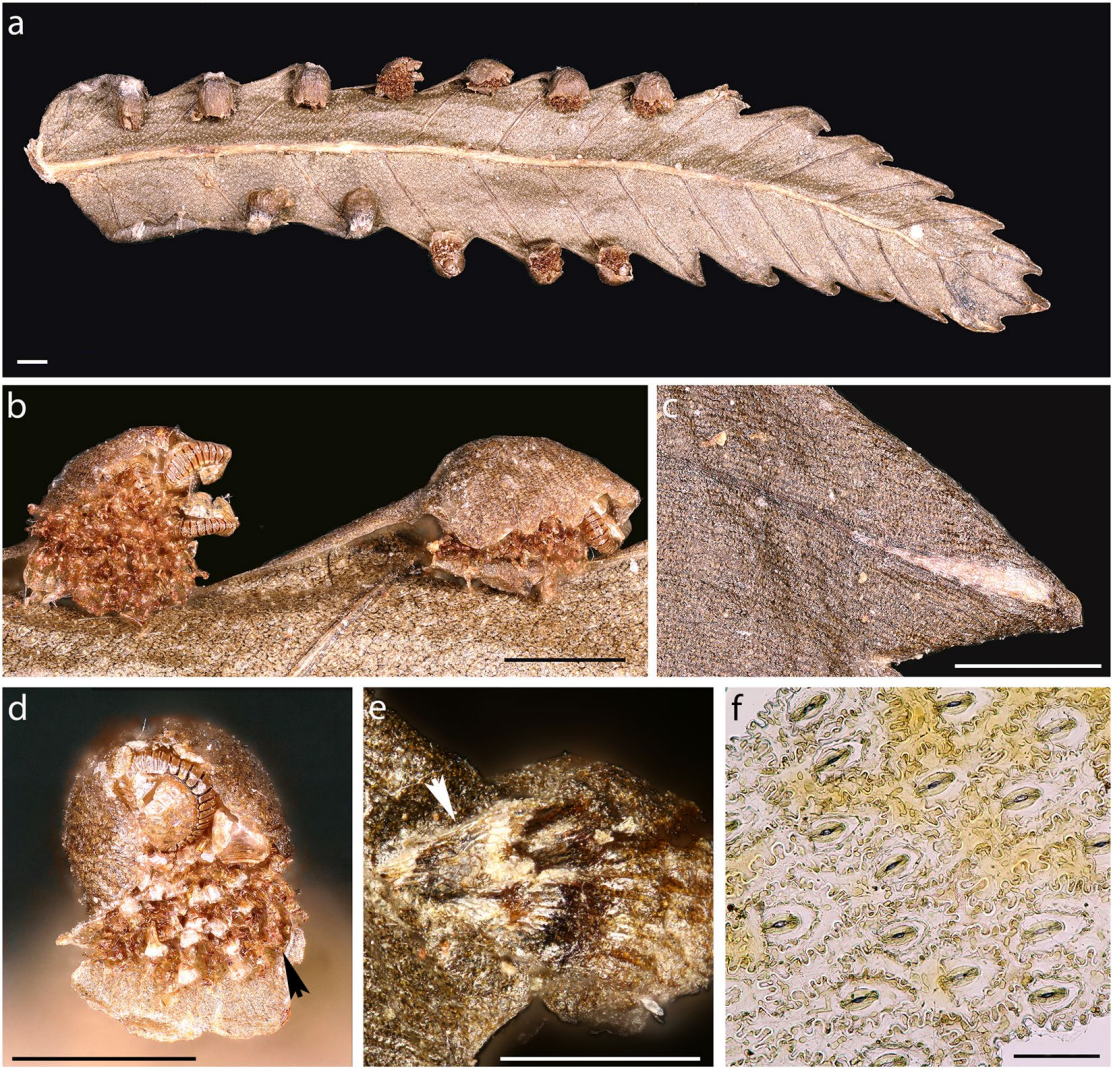
Leaf fragment of extant *Cystodium sorbifolium* (B 200138084). (**a**) Abaxial surface of fertile pinnule. (**b**) Sori viewed from abaxial side, showing abundant paraphyses. (**c**) Adaxial surface showing widened tips of veins. (**d**) Sorus showing outer indusium in upper part and inner indusium in lower portion; arrowhead indicates paraphyses. (**e**) Adaxial surface. White arrowhead shows area of polygonal epidermal cells at basis of outer indusium. (**f**) Abaxial epidermis showing polocytic stomata and epidermal cell with prominently sinuous anticlinal cell walls. Scale bars: 500 µm (**a**–**e**), 100 µm (**f**).

The only remaining repository for the fossil within Polypodiales is the Cystodiaceae, a monogeneric taxon comprised of the monotypic genus *Cystodium*^15,16,19^. The sole extant species in this genus, *C. sorbifolium*, shares with the fossil several key morphological features (Fig. 4), including (1) pinna axes with a prominent adaxial groove; (2) catadromous lamina division; (3) fertile pinnules with pronounced teeth protecting the sori (Figs 1d, 4a,b); (4) pinnule veins that are pinnately arranged, free, simple or forked; (5) submarginal sori positioned terminal on the veins; (6) a short receptacle that is slightly raised, round in cross-section, and protected by a reflexed lobe of the lamina (outer or false indusium); (7) a small and delicate, inner indusium that is attached at the base of the receptacle (Figs 1e,f, 4b,d); (8) sporangia with an annulus that is slightly oblique to almost longitudinal and interrupted by the pedicel (Fig. 1g); (9) numerous simple, multiseptate, non-glandular paraphyses interspersed with the sporangia (Figs 1f, 4b,d); and (10) trilete spores (Fig. 1h) that are globose and possess a low-sculptured perine^1,2,15,30,38^. The epidermis composed of polygonal cells seen in the outer indusium of *Cystodium sorbifolium* is yet another feature that the extant taxon shares with the fossil (Figs 1d,e, 4e). Taken together, the unique complement of morphological correspondences between the extant *C. sorbifolium* and the fossil renders *Cystodium* the only reasonable repository for the fossil. The fossil is largely identical morphologically to the extant *C. sorbifolium*, but there are also several minor but decisive structural differences that prompted us to introduce a new species, *Cystodium sorbifolioides*, for the fossil, rather than simply assigning the specimen to the extant *C. sorbifolium*.

*Cystodium sorbifolium* is characterized by hypostomatic leaves, and an adaxial epidermis composed of elongate cells with weakly undulate anticlinal walls that are regularly arranged parallel to the main vein, precisely as seen in the fossil (Figs 2a; Fig. 4 in^14^). On the other hand, the abaxial epidermis of *C. sorbifolium* differs markedly from that of the fossil because it is constructed of cells that usually are as long as wide and have prominently undulating anticlinal walls (Fig. 4f; Fig. 5 in^14^). Moreover, polocytic stomata, which are a typical feature of *C. sorbifolium* (Fig. 4f), have not been observed in the fossil (Fig. 2b,d). However, the preserved fragments of abaxial epidermis of *C. sorbifolioides* are small, and we therefore cannot rule out the possibility that polocytic stomata occurred somewhere in the fossil. Unfortunately, comparison of the fossil spore morphology and ornamentation with the granular-striate pattern seen in the spores of *C. sorbifolium*^30,38^ is impossible because all fossil spores are ill preserved and probably have been altered during embedding and fossilization in the amber matrix.

Discovery of a fossil representative of the genus *Cystodium* enables us to obtain a minimum age estimate for the Cystodiaceae and their relatives, the monogeneric Lonchitidaceae and the Lindsaeaceae. Lindsaeaceae fossils that have previously been described in the Burmese amber^22^ and provided support to the hypothesis that this lineage of ferns diversified before the Late Cretaceous. This result concurs with the age estimates obtained in previous studies for these lineages^11–13^. However, our analyses are not designed to obtain a maximum age estimate for the diversification of these lineages. In this context, it is important to note that the position of the Saccolomataceae remains incompletely resolved; it might be sister to all extant polypodialean lineages or sister to the Lindsaeineae^12,17,18^.

Together with the other ferns described to date in Burmese amber, the fern fossil detailed in this study suggests that, during the mid- to late Cretaceous, a fern flora existed in this region of the world that was composed mainly of representatives of families which still today occur in the lowland forests of SE Asia and Malesia, including Cystodiaceae, Dennstaedtiaceae^21^, and Lindsaeaceae^22^. The dennstaedtioid fossil *Krameropteris* H. Schneid. & al. is most similar to the extant genus *Monachosorum* that exclusively occurs in SE Asia^21,36^, while the Lindsaeaceae show a pantropical geographical distribution^39^. Eupolypod ferns have also been reported in Burmese amber, including *Cretacifilix* G.O. Poinar & R. Buckley^20^ and *Holttumopteris* L. Regalado & al.^23^. Given the restricted present-day distribution of *Cystodium* in tropical lowland forests in Malesia and part of Melanesia, the fossil *C. sorbifolioides* supports the hypothesis that the tropical forests in this region represent refugia for many tropical plant lineages that originated in the Cretaceous.

## Methods

Burmese amber comes from several localities near the village of Tanai in Kachin State, Myanmar^24,27^. Biostratigraphic studies^25^ and U-Pb dating of zircons^26^ suggest a late Albian to earliest Cenomanian age of Burmese amber, with a minimum age of 98 Ma.

Amber block (4.2 cm long, 1.9 cm wide) GZG.BST.21964 from the amber collection of the Geoscientific Collections of the University of Göttingen was studied using a Zeiss Stemi 2000 dissection microscope and a Zeiss AxioScope A1 compound microscope. After initial inspection, the block containing the fossil was cut into three portions (GZG.BST.21964 a to c) to come closer to the individual inclusions. Each portion was then ground and polished close to the inclusions using a series of wet silicon carbide papers (grit from FEPA P 600–4000, Struers) to minimize light scattering during subsequent detailed analysis and photographic documentation. Images were captured with a Canon EOS 5D digital camera attached to a compound microscope; incident and transmitted light were used simultaneously. To enhance the depiction of three-dimensional structures figures have been prepared as photomicrographic composites, digitally stacked from up to 60 focal planes using the software package HeliconFocus 6.7. Images of the extant *Cystodium sorbifolium* (B 200138084 (B), collected by C. E. Carr in 1935, in Papua New Guinea, forest at 1599 feet near Koitaki) were produced in Panorama 3D using a Keyence VHX-5000 Microscope. In order to prevent degradation of the amber specimens over time, the three amber specimens were embedded in a high-grade epoxy (EPO-TEK 301-2, Epoxy Technology, ratio of 100 [resin]: 37 [hardener]) under vacuum^40^.

A possible classification of the fossil was assessed using morphological evidence and comparisons to descriptions of extant fern families and genera in the literature^41^. Taxonomical updates were considered^19^.

The impact of the newly described fossil, as well as that of an unnamed Lindsaeaceae fossil^22^ on minimum age estimates was tested using a dated phylogenetic hypothesis for the putative extant relatives of the suborders Saccolomatineae and Lindsaeineae^19^. This hypothesis was derived from published DNA sequences of five plastid markers (*atpA*, *atpB*, *rbcL*, *trnH*-*psbA* and *trnL*-*trnF*) downloaded from Genbank (see Supplementary Material Table S1). The sequences were aligned manually using BioEdit 7.0.5.3^42^ and the chronogram was implemented in BEAST 1.8.2^43^. The GTR + I + G model was chosen as the best-fitting nucleotide substitution model in PARTITIONFINDER^44^ employing the Akaike Information Criterion. We tested three approaches generated using a lognormal relaxed clock: (1) the amber fossil here described placed as minimum age of the split of the Cystodiaceae and Lonchitidaceae-Lindsaeaceae lineages; (2) the unnamed Lindsaeaceae fossil^22^ conservatively considered a stem member of the family, thus assigned as minimum age of the split between Lonchitidaceae and Lindsaeaceae; and (3) an approach using both fossils. Calibrations were incorporated adopting a lognormal distribution with a minimum age set to 98 Ma^26^ and a truncate range of 98–400 Ma, with a birth-death prior accounting for incomplete sampling^45^, and a single partition for all five markers. Analyses were run for 80 million generations sampling every 8,000 generations. The results were inspected in TRACER 1.5^46^ and ESS values > 200 indicated ample mixing of the MCMC and a sufficient number of generations. These hypotheses were compared with published dating^11–13^ for the clades of Saccolomatineae and Lindsaeineae.

### Data availability

All data analysed during this study are included in this published article and its Supplementary Information: Supplementary Table S1.

## Electronic supplementary material

Supplementary Dataset 1
