# Publisher Correction: A fossil species of the enigmatic early polypod fern genus *Cystodium* (Cystodiaceae) in Cretaceous amber from Myanmar

**DOI:** 10.1038/s41598-017-17990-y

**Published:** 2018-01-08

**Authors:** Ledis Regalado, Alexander R. Schmidt, Marc S. Appelhans, Bork Ilsemann, Harald Schneider, Michael Krings, Jochen Heinrichs

**Affiliations:** 1 Ludwig Maximilian University, Faculty of Biology, Department of Biology and Geobio-Center, Menzinger Straße. 67, 80638 Munich, Germany; 2 Instituto de Ecología y Sistemática, Carretera de Varona 11835 e/ Oriente y Lindero, La Habana 19, CP 11900 Calabazar, Boyeros, La Habana Cuba; 3 University of Göttingen, Department of Geobiology, Goldschmidtstraße 3, 37077 Göttingen, Germany; 4 University of Göttingen, Albrecht-von-Haller Institute for Plant Sciences, Department of Systematics, Biodiversity and Evolution of Plants, Untere Karspuele 2, 37073 Göttingen, Germany; 5 SNSB-Bayerische Staatssammlung für Paläontologie und Geologie, Richard-Wagner-Straße 10, 80333 München, Germany; 6 Center for Integrative Conservation, Xishuangbanna Tropical Botanical Garden, Menglun, Mengla, 666303 Yunnan, China; 7 Natural History Museum, Department of Life Science, London, SW75BD UK; 8 Ludwig Maximilians University, Department of Earth and Environmental Sciences, Palaeontology and Geobiology, Richard-Wagner-Straße 10, 80333 München, Germany

Correction to: *Scientific Reports* 10.1038/s41598-017-14985-7, published online 03 November 2017

The original HTML version of this Article contained a typographical error in the publication date ‘03 November 2017’ which was incorrectly given as ‘06 November 2017’. This has now been corrected in the HTML version of the Article.

