# Publisher Correction: A fossil species of the enigmatic early polypod fern genus *Cystodium* (Cystodiaceae) in Cretaceous amber from Myanmar

**DOI:** 10.1038/s41598-018-22373-y

**Published:** 2018-03-06

**Authors:** Ledis Regalado, Alexander R. Schmidt, Marc S. Appelhans, Bork Ilsemann, Harald Schneider, Michael Krings, Jochen Heinrichs

**Affiliations:** 1 Ludwig Maximilian University, Faculty of Biology, Department of Biology and Geobio-Center, Menzinger Straße. 67, 80638 Munich, Germany; 2 Instituto de Ecología y Sistemática, Carretera de Varona 11835 e/ Oriente y Lindero, La Habana 19, CP 11900, Calabazar Boyeros, La Habana Cuba; 3 University of Göttingen, Department of Geobiology, Goldschmidtstraße 3, 37077 Göttingen, Germany; 4 University of Göttingen, Albrecht-von-Haller Institute for Plant Sciences, Department of Systematics, Biodiversity and Evolution of Plants, Untere Karspuele 2, 37073 Göttingen, Germany; 5 SNSB-Bayerische Staatssammlung für Paläontologie und Geologie, Richard-Wagner-Straße 10, 80333 München, Germany; 6 Center for Integrative Conservation, Xishuangbanna Tropical Botanical Garden, Menglun, Mengla, 666303 Yunnan China; 7 Natural History Museum, Department of Life Science, London, SW75BD UK; 8 Ludwig Maximilians University, Department of Earth and Environmental Sciences, Palaeontology and Geobiology, Richard-Wagner-Straße 10, 80333 München, Germany

Correction to: *Scientific Reports* 10.1038/s41598-017-14985-7, published online 03 November 2017

An error occurred after publication resulting in the inadvertent omission of the Article and Volume numbers from the PDF version of this Article. This error has now been corrected in the PDF version of the Article; the HTML version was correct from the time of publication.

