# Supplementary material for: A fossil species of the enigmatic early polypod fern genus Cystodium (Cystodiaceae) in Cretaceous amber from Myanmar
Source: Sci Rep. 2017 Nov 3;7:14615. doi: 10.1038/s41598-017-14985-7 (PMC5668433; doi:10.1038/s41598-017-14985-7)
Supplement: Supplementary file 1 — Supplementary Dataset 1 [file 41598_2017_14985_MOESM1_ESM.doc]

**Supplementary table for:**

**A fossil species of the enigmatic early polypod fern genus *Cystodium* (Cystodiaceae) in Cretaceous amber from Myanmar**

Ledis Regalado^1,2^, Alexander R. Schmidt^3^, Marc S. Appelhans^4^, Bork Ilsemann^5^, Harald Schneider^6,7^, Michael Krings^5,8^*, Jochen Heinrichs^1^

1 Ludwig Maximilian University, Faculty of Biology, Department of Biology and Geobio-Center, Menzinger Straße. 67, 80638 Munich, Germany

2 Instituto de Ecología y Sistemática, Carretera de Varona 11835 e/ Oriente y Lindero, La Habana 19, CP 11900, Calabazar, Boyeros, La Habana, Cuba

3 University of Göttingen, Department of Geobiology, Goldschmidtstraße 3, 37077 Göttingen, Germany

4 University of Göttingen, Albrecht-von-Haller Institute for Plant Sciences, Department of Systematics, Biodiversity and Evolution of Plants, Untere Karspuele 2, 37073 Göttingen, Germany

5 SNSB-Bayerische Staatssammlung für Paläontologie und Geologie, Richard-Wagner-Straße 10, 80333 München, Germany

6 Center for Integrative Conservation, Xishuangbanna Tropical Botanical Garden, Menglun, Mengla, 666303, Yunnan, China

7 Natural History Museum, Department of Life Science, London SW75BD, UK

8 Ludwig Maximilians University, Department of Earth and Environmental Sciences, Palaeontology and Geobiology, Richard-Wagner-Straße 10, 80333 München, Germany

* m.krings@lrz.uni-muenchen.de

Supplementary Table S1. Species and GenBank accession numbers of the DNA sequences used in this study.

| **Species** | **atpA** | **atp B** | **rbcL** | **trnH-psbA** |
| --- | --- | --- | --- | --- |
| *Cystodium sorbifolium* | EF463768 | AM184112 | AM184111 | HQ157299 |
| *Saccoloma inaequale* | EF463858 | EF463520 | EF463265 | JN575777 |
| *Saccoloma elegans* | - | HQ157279 | HQ157302 | GU478427 |
| *Lindsaea blotiana* | EF463769 | EF463476 | EF463230 | GU478511 |
| *Lindsaea multisora* | HQ157262 | HQ157278 | HQ157303 | GU478534 |
| *Lindsaea parasitica* | - | HQ157277 | LPU18640 | FJ360925 |
| *Lindsaea plicata* | HQ157271 | HQ157276 | HQ157304 | GU478471 |
| *Lindsaea quadrangularis* | EF463771 | EF463478 | EF463232 | FJ360929 |
| *Lonchitis hirsuta* | EF463772 | EU352277 | EU352305 | GU478429 |
| *Nesolindsaea kirkii* | HQ157265 | HQ157275 | HQ157307 | KC155835 |
| *Odontosoria aculeata* | EF463773 | EF463479 | EF463233 | GU478463 |
| *Odontosoria chinensis* | EF463774 | AY612710 | SCU05651 | AB575428 |
| *Osmolindsaea odorata* | HQ157264 | HQ157281 | LOU05630 | GU478431 |
| *Sphenomeris clavata* | HQ157267 | HQ157272 | HQ157301 | GU478448 |
| *Tapeinidium luzonicum* | HQ157266 | HQ157280 | HQ157300 | GU478444 |
